# Early Discontinuation of Opioid-Based Intravenous Patient-Controlled Analgesia After Single-Port Adnexal Laparoscopy: Incidence, Reasons, and Postoperative Outcomes

**DOI:** 10.3390/jcm15207774

**Published:** 2026-10-09

**Authors:** E Sun Paik, Yeji Jang, Hawon Kim, Jun-Hyeok Kang

**Affiliations:** 1 Department of Obstetrics and Gynecology, Kangbuk Samsung Hospital, Sungkyunkwan University School of Medicine, Seoul 03181, Republic of Korea; esun.paik@samsung.com (E.S.P.); yeji0422.jang@samsung.com (Y.J.); hawoni.kim@samsung.com (H.K.); 2 Department of Obstetrics and Gynecology, Uijeongbu Eulji Medical Center, Eulji University School of Medicine, Uijeongbu 11759, Republic of Korea

**Keywords:** single-port access laparoscopy, adnexal surgery, patient-controlled analgesia, postoperative nausea and vomiting, opioid-sparing analgesia, continuous wound infiltration

## Abstract

**Background/Objectives:** Opioid-based intravenous patient-controlled analgesia (IV-PCA) provides postoperative analgesia but can cause adverse effects that lead to early discontinuation. In relatively limited procedures such as single-port access (SPA) adnexal laparoscopy, the balance between analgesic benefit and opioid-related adverse effects remains uncertain. We evaluated the frequency of and recorded reasons for early IV-PCA discontinuation, associated factors, postoperative pain, and length of stay. **Methods:** We retrospectively reviewed 431 patients who received IV-PCA after single-port access (SPA) adnexal laparoscopy at two institutions. Early discontinuation was defined as permanent cessation before completion of the planned course. Associations with demographic and operative factors, concurrent continuous wound infiltration, and treating institution were examined. Repeated-measures analysis accounted for discontinuation timing and repeated pain assessments. **Results:** IV-PCA was discontinued early in 13.9% of patients. Among patients who discontinued IV-PCA, 85.0% had nausea or vomiting recorded as the reason for discontinuation. Of 59 patients with valid timing data, 47 (79.7%) discontinued within 24 h. No measured factor was significantly associated with discontinuation after adjustment. Unadjusted mean pain scores were 0.30, 0.39, and 0.28 points higher at 6, 12, and 24 h, respectively, but these differences were not significant after correction for multiple comparisons. Repeated-measures analysis showed no overall pain difference (+0.09 points, 95% CI −0.21 to +0.39). A secondary analysis suggested variation over time, but no time-specific estimate was statistically significant. Length of stay was similar between groups. **Conclusions:** Early IV-PCA discontinuation after SPA adnexal laparoscopy was mainly attributed to nausea or vomiting. These findings do not establish whether opioid-based IV-PCA is necessary or should be avoided in this setting; rather, they highlight the need to balance its potential analgesic benefit against opioid-related adverse effects and to prospectively evaluate alternative multimodal analgesic strategies.

## 1. Introduction

Single-port access (SPA) laparoscopy is widely used for benign adnexal disease [1,2]. An important advantage of SPA is that it limits surgical access to a single abdominal incision, thereby reducing the number of abdominal scars. However, this benefit must be balanced against the relatively large transumbilical incision required for the single-port approach [3], and surgical-site pain remains a clinically relevant concern [4,5]. Opioid-based intravenous patient-controlled analgesia (IV-PCA) provides effective postoperative analgesia [6]; however, whether routine use of a strong systemic opioid-based technique is necessary after relatively limited SPA adnexal surgery remains uncertain.

Opioids frequently cause systemic side effects—most commonly postoperative nausea and vomiting (PONV) and less often dizziness and hypotension [7,8]—that can lead to premature discontinuation of IV-PCA; previous studies have reported discontinuation in roughly 7–20% of patients [9]. This benefit–risk balance is particularly relevant in SPA adnexal surgery, which generally involves a relatively limited procedure and short operative time. Therefore, the potential analgesic benefit of opioid-based IV-PCA should be considered alongside its systemic adverse effects. Accordingly, contemporary guidelines increasingly emphasize multimodal, opioid-sparing analgesia after minimally invasive gynecologic surgery [10,11].

Despite this, the incidence, reasons, associated factors, and postoperative outcomes of IV-PCA discontinuation specifically after SPA adnexal laparoscopy have not been characterized. Notably, prior studies evaluating continuous wound infiltration (CWI) in this population excluded patients who discontinued IV-PCA [12,13], leaving this group unexamined. We therefore analyzed early IV-PCA discontinuation after SPA adnexal laparoscopy to quantify its incidence and recorded reasons, explore factors associated with discontinuation, and evaluate postoperative pain and length of stay.

## 2. Materials and Methods

### 2.1. Study Design and Patients

We retrospectively reviewed patients who underwent SPA laparoscopy for adnexal disease and received IV-PCA for postoperative pain control at Kangbuk Samsung Hospital or Uijeongbu Eulji Medical Center between 1 March 2020 and 30 September 2023. Patients who underwent ovarian cystectomy or salpingo-oophorectomy and who started IV-PCA postoperatively were included. Patients who underwent concurrent gynecologic surgery (such as hysterectomy or myomectomy) or who required conversion to multiport laparoscopy or laparotomy were excluded. Single-port access and direct-puncture single-port approaches were regarded as a single single-port technique. This cohort was drawn from the same institutional source population as two previous analyses from our group [12,13], which compared CWI with IV-PCA for postoperative pain control. Among patients who received IV-PCA, both studies by design analyzed only those who completed the planned course and excluded those who discontinued it early. Accordingly, the present maintained group overlaps substantially with the IV-PCA recipients of those reports, whereas patients who discontinued IV-PCA fell outside the analytic population of both studies, so their characteristics and postoperative course were not analyzed in either. This statement is based on the published exclusion criteria of those studies rather than on a patient-by-patient comparison; recruitment periods, patient flow, and the extent to which overlap could and could not be verified are set out in Table A2.

### 2.2. Ethical Aspects

This study was conducted in accordance with the Declaration of Helsinki and approved by the Institutional Review Boards of the Kangbuk Samsung Hospital (IRB No. 2022-12-030, IRB approval date: 21 December 2023) and Uijeongbu Eulji Medical Center (IRB No. 2023-02-015, IRB approval date: 13 December 2023). Because the study is a retrospective review of medical records, the approval dates follow the end of the treatment period covered by the review. The IRBs of both institutions waived the requirement for informed consent due to the retrospective nature of the study design.

### 2.3. Outcomes and Definitions

Patient demographic variables including age, body mass index (BMI), history of abdominal surgery, and treating institution were collected. Surgical outcome-related variables were total operation time, serum hemoglobin (Hb)-level change between preoperative and postoperative day 1, duration of hospital stay (from operation day to discharge day), and pathologic diagnosis.

The primary outcome was early, permanent discontinuation of IV-PCA before discharge, defined as cessation of the infusion before the prescribed course was complete and without restarting it during hospitalization. Patients whose IV-PCA ran to its planned completion or was removed as part of routine care and who therefore received the intended course comprised the maintained group. The decision to discontinue IV-PCA early was made by the treating clinical team, in most cases on the basis of clinical assessment of opioid-related side effects and in a minority at the patient’s request; the same clinical criteria were applied at both institutions, although no formal severity-scoring instrument was used and the records do not document the management steps that preceded each decision. The reason recorded at the time of discontinuation was categorized as nausea or vomiting, hypotension, dizziness, or other; the “other” category comprised four patients in whom a non-specific reason (for example, patient request or general discomfort) was recorded without a specific side effect. Postoperative surgical-site pain was assessed with the numeric rating scale (NRS, 0–10) [14] at 6, 12, 24, and 48 h, and length of hospital stay was defined as the interval from operation day to discharge.

### 2.4. Pain Management and Surgical Procedure

The postoperative pain management protocol was identical at the two institutions and has been described previously [12,13]. All patients received fentanyl-based IV-PCA immediately after surgery. According to age and body weight, 900–1800 μg of fentanyl citrate was diluted in 100 mL of 0.9% normal saline and infused at a basal rate of 1 mL/h, with a 15 μg bolus dose and a 15-min lockout interval. A rescue antiemetic (intravenous metoclopramide) was administered for postoperative nausea and vomiting of grade 2 or higher or at the patient’s request; nausea and vomiting were graded on the four-point scale used in our previous analyses of this population (0, no nausea; 1, mild nausea; 2, severe nausea; 3, vomiting) [12,13]. This grading governed the administration of rescue antiemetics only and was not used to decide whether to discontinue IV-PCA. After resumption of an oral diet, oral NSAIDs were given twice daily until discharge (oral acetaminophen in cases of NSAID allergy); for breakthrough pain, NSAIDs were administered first and opioids only when necessary. In patients who additionally received continuous wound infiltration, an elastomeric pump (ON-Q PainBuster; I-Flow, LLC, Irvine, CA, USA) delivered 100 mL of 0.3% ropivacaine at 2 mL/h through a multi-hole catheter positioned on the musculofascial layer of the umbilical wound.

All operations were single-port access laparoscopy performed through a 2.0–2.5 cm transumbilical incision with a single-port wound retractor (Glove Port; Nelis Co., Ltd., Bucheon, Republic of Korea) and CO_2_ pneumoperitoneum (12 mmHg); ovarian cystectomy or salpingo-oophorectomy was performed, and specimens were retrieved using an in-bag technique, as described previously [12,13].

### 2.5. Statistical Analysis

Continuous variables were summarized as mean ± standard deviation or median [interquartile range] based on the Shapiro–Wilk test and compared using the Mann–Whitney U test; categorical variables were summarized as number (percentage) and compared using the chi-square or Fisher’s exact test. The 95% confidence interval for the discontinuation rate was calculated by the Wilson score method. No statistical analysis plan was registered before the study, but the covariates for the multivariable model were specified before it was fitted, on clinical grounds rather than by data-driven selection: age, body mass index, previous abdominal surgery, adnexal procedure type, operative time, and concurrent CWI. Treating institution was added, and missing body mass index was handled by multiple imputation rather than complete-case analysis post hoc.

Factors associated with discontinuation were assessed with multivariable logistic regression using a Firth-penalized likelihood approach, which reduces small-sample bias given the limited number of events (*n* = 60). Body mass index was missing in 12 of 431 patients (2.8%), and the missingness was related to both the outcome and the treating institution (Section 3.2); it was therefore handled by multiple imputation by chained equations (predictive mean matching, 20 imputed datasets, with convergence checked; R package mice), with the outcome and all model covariates included in the imputation model, and the Firth estimates were pooled using Rubin’s rules. This assumes that the data are missing at random given the observed variables, an assumption that cannot be verified and on which the institution estimate particularly depends; a complete-case analysis is therefore reported as a sensitivity analysis (Section 3.2 and Table A3). Histologic diagnosis was excluded because several subgroups (e.g., ovarian cancer, borderline tumor) were too sparse for stable estimation, and intraoperative opioid dose was not available as a structured variable.

Postoperative NRS scores at each time point and length of stay were compared between groups using the Mann–Whitney U test; nonparametric tests were used given the ordinal nature of the NRS, although NRS values are presented as mean ± SD for consistency with the figures. These timepoint-wise comparisons and the post hoc comparison stratified by concurrent CWI use were unadjusted, did not model within-patient correlation, were not corrected for multiple testing, and are therefore reported as exploratory. A two-sided *p*-value < 0.05 was considered statistically significant.

To address the repeated-measures structure of the pain data and the fact that discontinuation is a post-baseline, time-varying event, we additionally fitted generalized estimating equation (GEE) models to the NRS assessments in long format (Gaussian family with identity link, exchangeable working correlation, robust sandwich standard errors, patient as the clustering unit). The exposure was time-varying and was defined as whether early discontinuation had already occurred before that assessment; because the time at which IV-PCA ended in patients who completed the planned course was not recorded, this variable indexes prior early discontinuation rather than verified receipt of IV-PCA at the moment of assessment. The models included assessment time and concurrent CWI, with a secondary specification adjusted for age and institution, a comparison model using the time-fixed “ever discontinued” classification, and a test of the interaction between discontinuation status and assessment time. All models used the same dataset: of the 1564 available NRS assessments in 431 patients, three assessments from the patient with an out-of-range timing record were excluded because exposure status could not be determined, and one patient contributed no assessment, leaving 1561 observations from 429 patients. Only observed assessments were used; missing NRS values were neither imputed nor weighted. Analyses were performed using R version 4.4.2 (R Foundation for Statistical Computing, Vienna, Austria), with the packages tableone, mice, logistf, and geepack.

## 3. Results

### 3.1. Incidence and Reasons for IV-PCA Discontinuation

**Figure 1:**
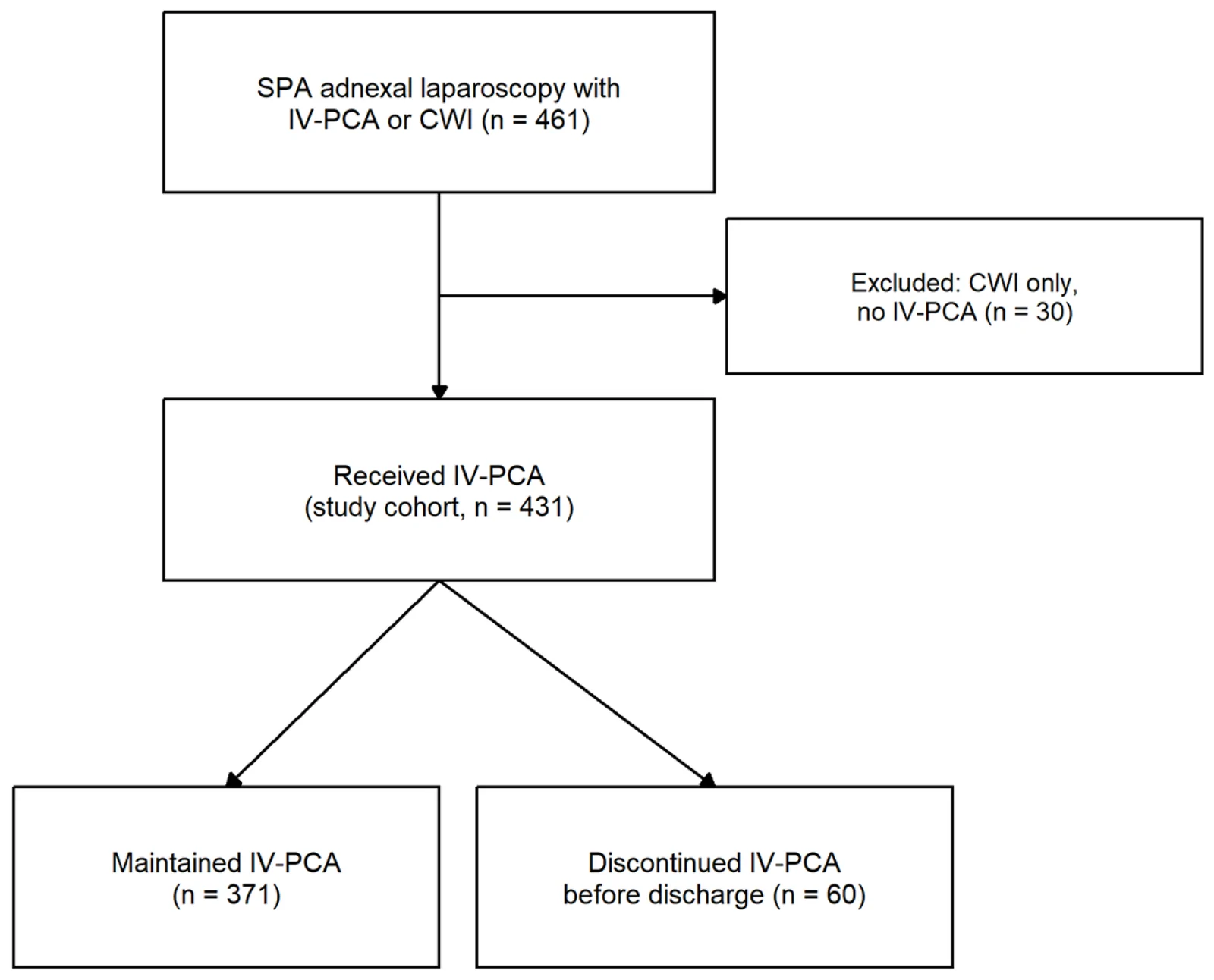
Patient selection. SPA, single-port access; IV-PCA, intravenous patient-controlled analgesia; CWI, continuous wound infiltration.

**Figure 2:**
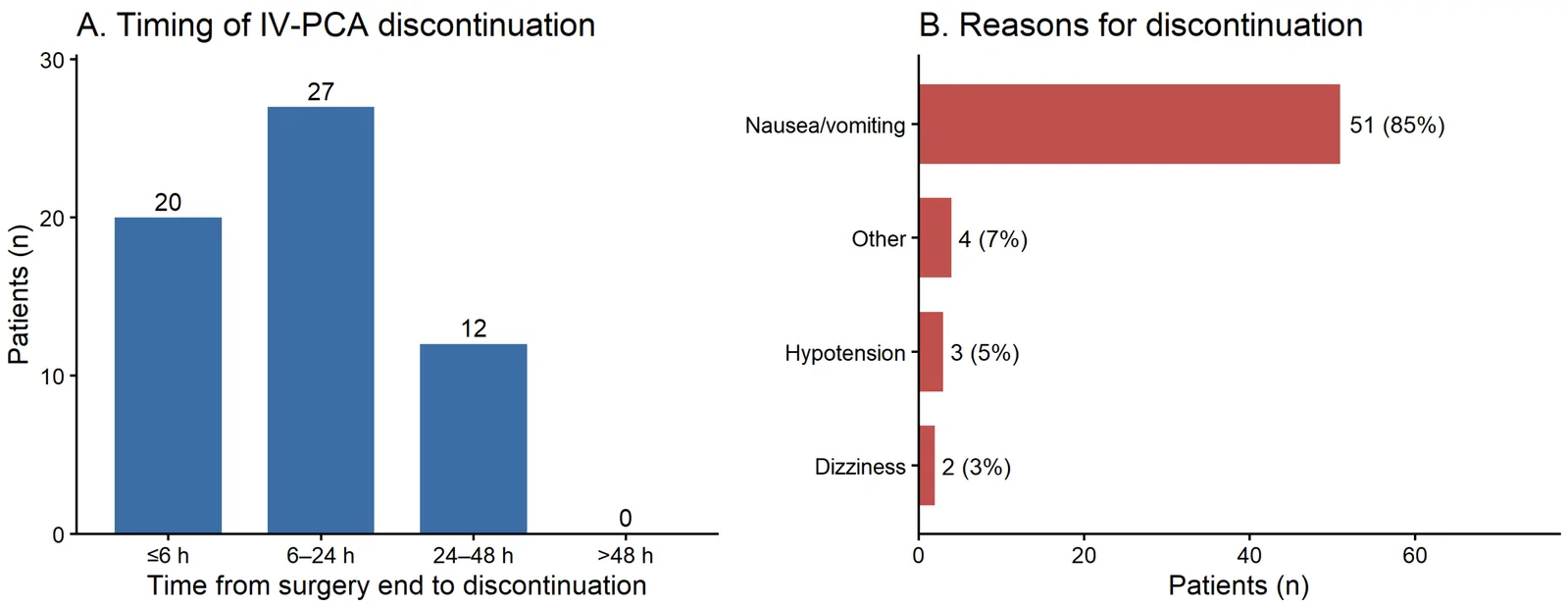
Timing and reasons for IV-PCA discontinuation. (**A**) Timing of IV-PCA discontinuation, expressed as the interval from the end of surgery; categories are ≤6 h, >6–24 h, >24–48 h, and >48 h. (**B**) Reasons for discontinuation. Most discontinuations occurred within 24 h and were attributable to nausea or vomiting. One of the 60 discontinuers was excluded from panel A because the recorded time of IV-PCA clamping preceded the recorded end of surgery, an internally inconsistent timestamp that could not be resolved from the medical record; that patient is included in the descriptive analyses and in the analysis of factors associated with discontinuation but is excluded from all generalized estimating equation models so that the same analytic dataset is used across models (Section 2.5).

The 431 patients had a mean age of 38.8 ± 13.9 years and a mean body mass index of 23.4 ± 4.0 kg/m^2^. The most common pathologic diagnoses were other benign conditions (37.8%) and endometriosis (35.0%); 143 patients (33.2%) underwent salpingo-oophorectomy rather than cystectomy, and 188 (43.6%) received concurrent CWI (Table 1). Of 431 patients who started IV-PCA after SPA adnexal laparoscopy, 60 (13.9%; 95% CI 11.0–17.5%) discontinued it before discharge, whereas 371 (86.1%) completed the planned course without early discontinuation (Figure 1). Discontinuation occurred in 50 of 389 patients (12.9%) at Kangbuk Samsung Hospital and in 10 of 42 patients (23.8%) at Uijeongbu Eulji Medical Center (χ^2^ *p* = 0.087). The predominant reason for discontinuation was nausea or vomiting (51/60, 85.0%), followed by hypotension (3/60, 5.0%), dizziness (2/60, 3.3%), and other causes (4/60, 6.7%). Among the 59 discontinuers with a valid discontinuation-timing record, most discontinuations occurred early, within 24 h of the end of surgery, and none occurred after 48 h (Figure 2).

### 3.2. Factors Associated with Discontinuation

Baseline characteristics of the two groups are presented in Table 1. Patients who discontinued IV-PCA were significantly older than those who maintained it (42.7 ± 13.7 vs. 38.2 ± 13.8 years, *p* = 0.011). No other baseline or operative variable differed significantly between groups, although discontinuers showed non-significant trends toward a higher rate of previous laparotomy (30.0% vs. 19.4%, *p* = 0.089) and oophorectomy rather than cystectomy (43.3% vs. 31.5%, *p* = 0.098). Concurrent CWI was similarly distributed between the discontinued and maintained groups (48.3% vs. 42.9%, *p* = 0.514), as was the distribution of patients across the two institutions (Table 1). In the multivariable Firth-penalized logistic regression (Table 2), which included age, body mass index, previous abdominal surgery, adnexal procedure type, operative time, concurrent CWI, and treating institution, no statistically significant association with discontinuation was detected for any covariate; the univariate association with age was attenuated after adjustment (adjusted OR 1.02 per year, 95% CI 0.99–1.05, *p* = 0.136). In the complete-case sensitivity analysis (*n* = 419, 51 events; Table A3), the estimates for the clinical covariates were broadly similar and none was statistically significant, but the estimate for institution differed substantially and was in the opposite direction (OR 0.29, 95% CI 0.03–1.23). This instability corresponds to the loss, through missing body mass index data, of 9 of the 10 discontinuation events contributed by the smaller institution, and the two analyses should therefore be read together rather than as one superseding the other.

### 3.3. Postoperative Outcomes Associated with Discontinuation

**Figure 3:**
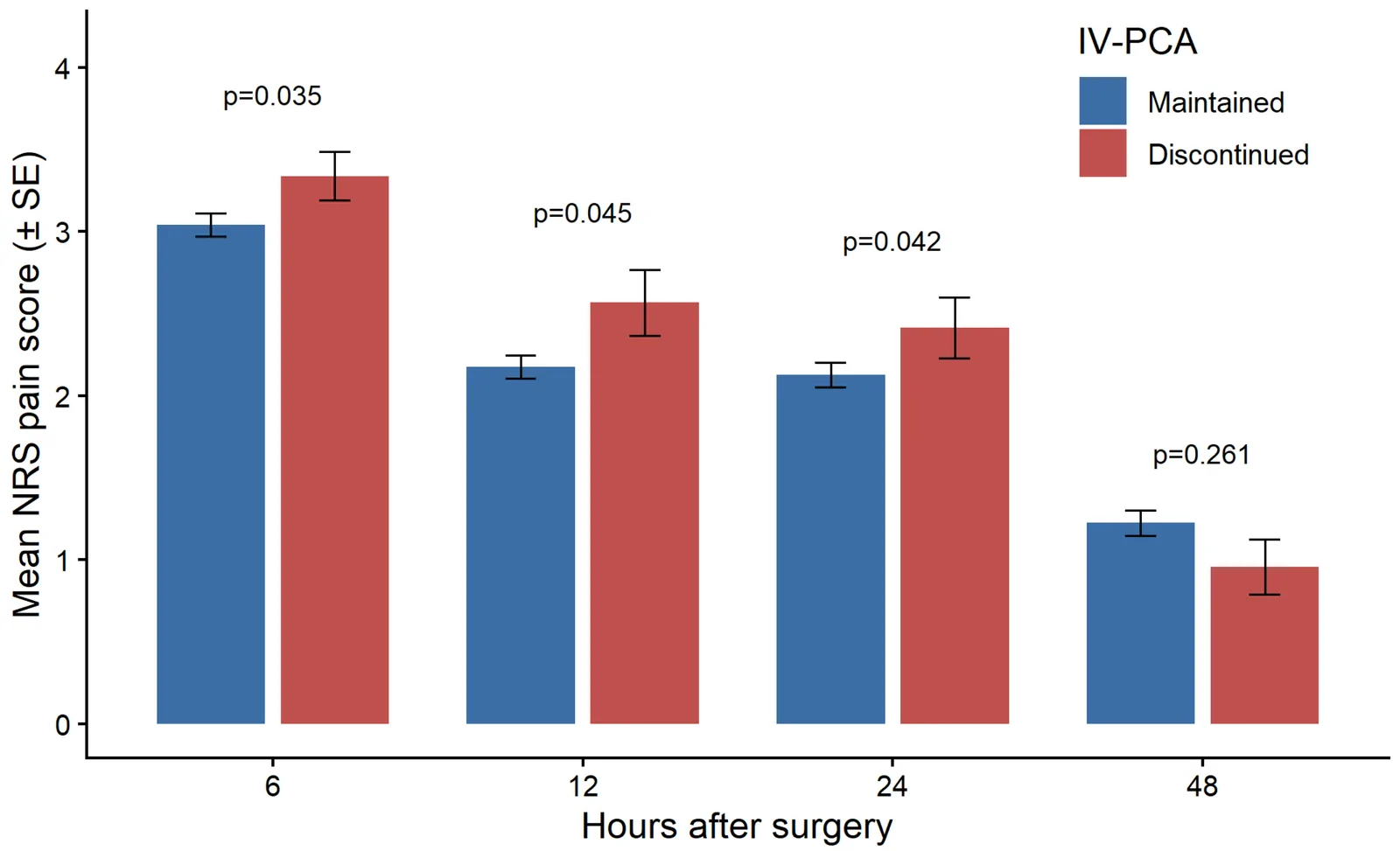
Postoperative pain by IV-PCA status. Mean numeric rating scale (NRS) pain scores (±standard error) at 6, 12, 24, and 48 h in patients who maintained versus discontinued IV-PCA. *p*-values are from the Mann–Whitney U test and are unadjusted and uncorrected for multiple testing; these comparisons are exploratory. Mean NRS scores were higher in the discontinuation group at 6, 12, and 24 h and converged to a near-floor level in both groups by 48 h.

Patients who discontinued IV-PCA had higher postoperative pain scores during the first 24 h (Table 3, Figure 3): NRS scores were higher at 6 h (3.34 vs. 3.04, *p* = 0.035), 12 h (2.57 vs. 2.18, *p* = 0.045), and 24 h (2.41 vs. 2.13, *p* = 0.042). These comparisons were unadjusted and uncorrected for multiple testing; none of the three would remain significant under a Bonferroni-corrected threshold for four time points (α = 0.0125), and they should therefore be interpreted as exploratory. By 48 h, pain had declined to a near-floor level in both groups (median NRS 1, with approximately 40% of patients reporting no pain) and did not differ between groups (0.96 vs. 1.22, *p* = 0.261). Length of hospital stay was also similar (median 3 days; *p* = 0.453). The number of patients with an available NRS assessment at each time point (numbers at risk) is reported in the footnote to Table 3.

### 3.4. Sensitivity Analysis According to the Timing of Discontinuation

Because the timing of discontinuation varied between patients, we performed a sensitivity analysis restricted to NRS assessments obtained after IV-PCA discontinuation (Table A1). Among discontinuers who were still receiving IV-PCA at the 6-h assessment, pain did not differ from the maintained group (3.24 vs. 3.04, *p* = 0.217). When comparisons were limited to post-discontinuation assessments, the difference at 12 h (2.69 vs. 2.18, *p* = 0.087) and at 24 h (2.37 vs. 2.13, *p* = 0.162) was no longer statistically significant, and the difference at 48 h (0.96 vs. 1.22, *p* = 0.261), which had not been significant in the unadjusted comparison either, remained non-significant. This does not indicate that the differences became smaller: at 12 h the mean difference was larger in the restricted comparison (+0.52 points, *n* = 26) than in the unadjusted comparison (+0.39 points, *n* = 51), whereas at 24 h it was slightly smaller (+0.24 vs. +0.29 points); these differences are calculated from unrounded means and the restricted subgroups differ from the unadjusted comparison in size, composition, and variance, so the change in statistical significance should not be attributed to any single cause. Patients who discontinued within 6 h had higher pain at 12 h (3.00 vs. 2.18, *p* = 0.008), a difference that was no longer apparent at 24 h (*p* = 0.351). In a further post hoc analysis stratified by concurrent CWI use, the between-group difference at 6 h reached nominal significance in the stratum without CWI (3.50, *n* = 30 vs. 3.08, *n* = 212; *p* = 0.038) but not in the CWI stratum (3.17, *n* = 29 vs. 2.99, *n* = 159; *p* = 0.336); the remaining time points were not significant in either stratum (Table A4). All of these subgroups are small, and these analyses are exploratory and hypothesis-generating rather than confirmatory.

### 3.5. Repeated-Measures Analysis with Time-Varying Discontinuation Status

The GEE analyses comprised 1561 NRS assessments from 429 patients (estimated within-patient correlation 0.19); the derivation of these numbers is given in Section 2.5. In the model treating discontinuation as a time-varying exposure and adjusted for assessment time and concurrent CWI, no statistically significant difference in NRS score was detected between assessments made after early discontinuation and those made before it (mean difference +0.09 points, 95% CI −0.21 to +0.39, *p* = 0.548). The estimate was similar after additional adjustment for age and institution (+0.17 points, 95% CI −0.12 to +0.45, *p* = 0.250), and a model using the time-fixed “ever discontinued” classification was likewise non-significant (+0.17 points, 95% CI −0.05 to +0.40, *p* = 0.123); all three models were fitted to the same 1561 observations from 429 patients (Table 4). Because a single coefficient for discontinuation status assumes a difference that is common to all assessment times, we also tested, as a post hoc analysis, an interaction between discontinuation status and assessment time; this interaction was statistically significant (Wald χ^2^ = 9.46, df = 3, *p* = 0.024), indicating that the difference associated with prior early discontinuation varied across assessment times. The time-specific estimates were +0.33 (95% CI −0.21 to +0.87) at 6 h, +0.53 (−0.06 to +1.12) at 12 h, +0.23 (−0.21 to +0.67) at 24 h, and −0.34 (−0.71 to +0.03) at 48 h; none of these differed significantly from zero individually, and the pattern of positive estimates during the first 24 h and a negative estimate at 48 h is consistent with the significant interaction. In summary, the models assuming a common difference did not detect a statistically significant association between prior early discontinuation and NRS score, whereas the exploratory interaction analysis suggests that any such association may vary over time; the time-specific estimates are imprecise, and because this interaction was one of several post hoc tests, it requires confirmation in an independent dataset.

## 4. Discussion

In this two-center cohort of patients undergoing SPA adnexal laparoscopy, 13.9% (95% CI 11.0–17.5%) discontinued opioid-based IV-PCA before discharge, the great majority because of nausea or vomiting, and most within 24 h of surgery. No statistically significant association with discontinuation was detected among the clinical covariates analyzed, including treating institution. Patients who discontinued IV-PCA had higher observed pain scores during the first 24 postoperative hours in unadjusted comparisons; however, these were uncorrected for multiple testing, and a repeated-measures model assuming a common difference across assessment times did not detect a significant difference, although a post hoc interaction suggested that any association may vary over time. Hospital stay did not differ between groups.

The 13.9% discontinuation rate observed here is consistent with the 7–20% range reported for opioid-based IV-PCA in prior studies [9]. Nausea or vomiting was recorded as the reason for discontinuation in 51 of 60 patients (85.0%), consistent with the well-recognized adverse-effect profile of opioids [7,8,15]. Importantly, this percentage represents the recorded reasons among patients who discontinued IV-PCA and should not be interpreted as the incidence of nausea or vomiting in the entire cohort. Because all patients in the present study underwent SPA surgery and no conventional multiport comparison group was included, our data cannot determine whether SPA is associated with distinct postoperative analgesic requirements or whether the observed discontinuation pattern is specific to the single-port approach. Furthermore, actual opioid exposure or consumption was not available in sufficient detail to assess whether opioid-related adverse effects were dose-dependent. Accordingly, we cannot determine whether patients with greater pain received more opioids and subsequently experienced more nausea or vomiting. Although nausea or vomiting was the predominant recorded reason for discontinuation, the available records do not allow us to determine whether discontinuation reflected adverse effects alone or a combination of adverse effects, pain severity, and the perceived need for continued opioid analgesia. Our findings extend prior work on this cohort: previous studies reported an analgesic and opioid-sparing benefit of adding CWI to IV-PCA and similar pain outcomes for CWI alone compared with IV-PCA outside the early postoperative period; however, among IV-PCA recipients, they analyzed only patients who maintained IV-PCA and excluded those who discontinued it, and the second study additionally included a group treated with CWI alone who never received IV-PCA [12,13]. The present study is complementary, focusing directly on the previously excluded group and characterizing an outcome—IV-PCA discontinuation itself—that those analyses did not address.

Two findings have practical implications. First, discontinuation was overwhelmingly attributed to nausea or vomiting in the recorded reasons. In the multivariable model, no statistically significant association with discontinuation was detected among the demographic and surgical variables examined. Given that several established determinants of postoperative nausea and vomiting were unavailable in this dataset (see below), this should be read as a limitation of the available variables rather than as evidence that such patients cannot be identified. Second, patients who discontinued IV-PCA had higher pain scores during the early postoperative period. The observational design and the exploratory, unadjusted nature of these comparisons preclude establishing whether discontinuation itself caused the higher scores, and repeated-measures models that treated discontinuation as a time-varying exposure did not detect a statistically significant difference in NRS score, either overall or at any individual assessment time (Section 3.5); the mean differences observed in the unadjusted timepoint-wise comparisons were modest (approximately 0.3–0.4 NRS points), although the confidence intervals around the time-specific estimates from the repeated-measures model were considerably wider (for example, +0.53, 95% CI −0.06 to +1.12 at 12 h), so the magnitude of any early difference is not established with precision. Because a substantial proportion of patients in both groups also received CWI (48.3% of discontinuers vs. 42.9% of those who maintained IV-PCA; Table 1), we compared pain scores stratified by concurrent CWI use (Section 3.4). The between-group difference reached nominal significance only in the stratum without CWI; with the available subgroup sizes we cannot determine whether this reflects effect modification by CWI, differing statistical power, or residual confounding, and CWI should therefore be regarded as an unresolved potential influence on this comparison. Alternative multimodal and opioid-sparing analgesic approaches have been investigated in postoperative care, including CWI [16] and strategies that omit IV-PCA in selected laparoscopic procedures [17]. However, the present study was not designed to compare analgesic strategies and therefore cannot establish the superiority or equivalence of CWI or any other alternative regimen.

Taken together, our findings should not be interpreted as showing that opioid-based IV-PCA is either necessary or unnecessary after SPA adnexal surgery. Rather, they indicate that when opioid-based IV-PCA is used in this setting, early discontinuation is not uncommon and is predominantly attributed to well-recognized opioid-related adverse effects. The clinically relevant question is therefore the balance between analgesic benefit and these adverse effects. Because the present study did not directly compare opioid-based IV-PCA with non-opioid or opioid-sparing strategies, the optimal postoperative analgesic approach remains uncertain and should be addressed in prospective comparative studies.

Patient-related risk factors for postoperative nausea and vomiting—such as a personal history of PONV or motion sickness, non-smoking status, and anxiety, several of which are components of established risk scores [18]—and perioperative medication and symptom-management data—including prophylactic antiemetic use, intraoperative opioid dose or fentanyl concentration, and rescue antiemetic administration—were not available as structured variables in the present dataset [18] and may better explain who discontinues IV-PCA; these represent important targets for prospective evaluation.

This study has several limitations. It was retrospective and non-randomized, and the choice of analgesic strategy was not controlled. Detailed rescue-analgesic data were not available for patients who discontinued IV-PCA, so the outcome analysis was limited to pain scores and length of stay. IV-PCA discontinuation is a post-baseline, time-varying event, whereas pain was compared at fixed assessment times: the timing of discontinuation varied between patients and, in most cases, followed the early assessments—39 of 60 patients discontinued IV-PCA more than 6 h after surgery—so the higher early pain scores in the discontinuation group cannot be attributed to discontinuation itself and may partly reflect pre-existing differences in pain. Our timepoint-wise comparisons were complete-case analyses that did not model within-patient correlation across repeated assessments and were not adjusted for multiple testing or for potential confounders; we therefore also fitted generalized estimating equation models treating discontinuation as a time-varying exposure (Section 3.5), which did not detect a statistically significant difference in pain; those models nonetheless remain observational and cannot exclude residual confounding, and they use the observed NRS assessments only, without imputation or weighting, so the analysis does not correct for any bias that a non-random missingness process could introduce. The post hoc interaction between discontinuation status and assessment time was one of several exploratory tests and the resulting time-specific estimates were imprecise, so it should be regarded as hypothesis-generating. A failure to detect a statistically significant difference is also not evidence that discontinuation has no effect on pain or that analgesia after discontinuation was equivalent. The sensitivity analysis restricted to post-discontinuation assessments (Table A1) is likewise limited, because its subgroups are defined by the timing of a future event and are small. Several established determinants of postoperative nausea and vomiting were unavailable as structured variables, as detailed above. The smaller of the two participating institutions contributed only 42 patients and 10 discontinuations, which limits the precision of the institution term in the multivariable model and precludes a meaningful institution-stratified analysis of secondary outcomes. Because all patients underwent SPA surgery and no multiport comparator was included, the present study cannot determine whether postoperative analgesic requirements or IV-PCA discontinuation differ between single-port and conventional multiport laparoscopy. Actual opioid consumption was also not available in sufficient detail, preventing assessment of a dose–response relationship between opioid exposure and adverse effects. In addition, although the recorded reason for discontinuation was available, the retrospective records did not permit us to determine whether discontinuation decisions reflected adverse effects alone or the combined influence of adverse effects, pain severity, and perceived analgesic need. Perioperative analgesic practices may also differ across institutions and countries; both participating institutions were Korean centers using a similar IV-PCA protocol, which may limit generalizability to other healthcare settings. The study was also limited to adnexal surgery, so the findings may not generalize to other SPA procedures with longer operative and specimen-retrieval times. Strengths include the analysis of a clinically meaningful, objectively defined endpoint (actual discontinuation rather than a surrogate) in a population that prior studies excluded.

## 5. Conclusions

In conclusion, approximately one in seven patients (13.9%; 95% CI 11.0–17.5%) discontinued opioid-based IV-PCA before discharge after SPA adnexal laparoscopy, predominantly because of nausea or vomiting, and most did so within 24 h of surgery. No statistically significant association with discontinuation was detected among the clinical covariates analyzed, although several established risk factors for postoperative nausea and vomiting could not be examined. Patients who discontinued IV-PCA had modestly higher early pain scores in exploratory, unadjusted comparisons, but repeated-measures analyses did not detect an overall difference, and an exploratory interaction suggested that any association may vary across assessment times. Whether these patients required additional analgesia was not evaluated. These findings do not establish whether opioid-based IV-PCA is necessary or should be avoided after SPA adnexal surgery. Rather, they show that when opioid-based IV-PCA is used in this setting, early discontinuation is not uncommon and is predominantly attributed to well-recognized opioid-related adverse effects. The potential analgesic benefit of opioid-based IV-PCA should therefore be considered alongside these adverse effects, and prospective studies directly comparing opioid-based IV-PCA with alternative multimodal analgesic strategies are warranted.

## Figures and Tables

**Table 1:** Baseline demographic and surgical characteristics according to IV-PCA status.

Characteristic	Total (*n* = 431)	Maintained (*n* = 371)	Discontinued (*n* = 60)	*p*-Value
Age (years)	38.79 ± 13.87	38.16 ± 13.81	42.68 ± 13.68	0.011 *
Body mass index (kg/m^2^) ^‡^	23.42 ± 4.00	23.49 ± 4.13	22.92 ± 2.92	0.783
Previous abdominal surgery	154 (35.7)	129 (34.8)	25 (41.7)	0.374
Previous laparoscopy	75 (17.4)	66 (17.8)	9 (15.0)	0.730
Previous laparotomy	90 (20.9)	72 (19.4)	18 (30.0)	0.089
Pathologic diagnosis				0.232
Teratoma	99 (23.0)	91 (24.5)	8 (13.3)	
Endometriosis	151 (35.0)	131 (35.3)	20 (33.3)	
Other benign	163 (37.8)	135 (36.4)	28 (46.7)	
Borderline ovarian tumor	12 (2.8)	9 (2.4)	3 (5.0)	
Ovarian cancer	6 (1.4)	5 (1.3)	1 (1.7)	
Oophorectomy (vs. cystectomy)	143 (33.2)	117 (31.5)	26 (43.3)	0.098
Bilateral procedure ^†^	127 (30.2)	105 (28.9)	22 (37.9)	0.217
Operative time (min)	31 [22–45]	31 [22–45]	32.5 [24.8–44.3]	0.541
Estimated blood loss (mL)	50 [30–50]	50 [30–50]	30 [30–52.5]	0.311
Hemoglobin change (g/dL)	1.95 ± 0.97	1.95 ± 0.96	1.97 ± 1.04	0.924
Concurrent CWI	188 (43.6)	159 (42.9)	29 (48.3)	0.514
Institution				0.087
Kangbuk Samsung Hospital	389 (90.3)	339 (91.4)	50 (83.3)	
Uijeongbu Eulji Medical Center	42 (9.7)	32 (8.6)	10 (16.7)	

Values are mean ± SD, median [IQR], or *n* (%), and all descriptive statistics are based on observed data. Continuous variables were compared using the Mann–Whitney U test and categorical variables using the chi-square or Fisher’s exact test. * *p* < 0.05. ^†^ Laterality could not be determined in 10 patients; percentages are based on non-missing values. ^‡^ Body mass index was recorded in 419 of 431 patients (maintained group, 368; discontinued group, 51); missing values were multiply imputed for the multivariable model only (Section 2.5). CWI, continuous wound infiltration; IQR, interquartile range; IV-PCA, intravenous patient-controlled analgesia; SD, standard deviation.

**Table 2:** Multivariable Firth-penalized logistic regression for IV-PCA discontinuation.

Variable	Adjusted OR	95% CI	*p*-Value
Age (per year)	1.02	0.99–1.05	0.136
Body mass index (per kg/m^2^)	0.96	0.89–1.04	0.306
Previous abdominal surgery	1.22	0.69–2.18	0.494
Oophorectomy (vs. cystectomy)	1.11	0.53–2.32	0.789
Operative time (per min)	1.00	0.99–1.02	0.671
Concurrent CWI	1.23	0.70–2.14	0.471
Institution: Uijeongbu Eulji Medical Center	2.00	0.85–4.74	0.115

Firth-penalized logistic regression fitted within 20 multiply imputed datasets (body mass index missing in 12 of 431 patients) and pooled using Rubin’s rules; all 431 patients and 60 events contributed. Reference categories: no previous abdominal surgery, cystectomy, no concurrent CWI, and Kangbuk Samsung Hospital. Histologic diagnosis and intraoperative opioid dose were not included (see Section 2.5). The univariate association with age (*p* = 0.011) was attenuated after adjustment. CI, confidence interval; CWI, continuous wound infiltration; IV-PCA, intravenous patient-controlled analgesia; OR, odds ratio.

**Table 3:** Postoperative pain and length of stay according to IV-PCA status.

Outcomes	Maintained (*n* = 371)	Discontinued (*n* = 60)	*p*-Value
NRS at 6 h	3.04 ± 1.37	3.34 ± 1.12	0.035 *
NRS at 12 h	2.18 ± 1.25	2.57 ± 1.43	0.045 *
NRS at 24 h	2.13 ± 1.39	2.41 ± 1.40	0.042 *
NRS at 48 h	1.22 ± 1.40	0.96 ± 1.17	0.261
Length of stay (days)	3 [3–3]	3 [3–3]	0.453

NRS values are mean ± SD; length of stay is median [IQR]. Groups were compared using the Mann–Whitney U test; these are unadjusted, exploratory comparisons that were not corrected for multiple testing. * Unadjusted *p* < 0.05. At 48 h, pain converged to a near-floor level in both groups. Numbers at risk (patients with an available NRS assessment) were: maintained group, 371, 300, 348, and 329 at 6, 12, 24, and 48 h, respectively; discontinued group, 59, 51, 58, and 48 at 6, 12, 24, and 48 h, respectively. Missing assessments were excluded pairwise from each comparison (complete-case analysis); no imputation was performed. IQR, interquartile range; IV-PCA, intravenous patient-controlled analgesia; NRS, numeric rating scale; SD, standard deviation.

**Table 4:** Generalized estimating equation models of postoperative NRS score according to IV-PCA discontinuation status.

Model	Exposure	Adjustment	Mean Difference	95% CI	*p*-Value
1 (primary)	Early discontinuation already occurred (time-varying)	Assessment time, concurrent CWI	+0.09	−0.21 to +0.39	0.548
2	Early discontinuation already occurred (time-varying)	Model 1 + age, institution	+0.17	−0.12 to +0.45	0.250
3	Ever discontinued (time-fixed)	Assessment time, concurrent CWI	+0.17	−0.05 to +0.40	0.123
4	Early discontinuation × assessment time	Assessment time, concurrent CWI	see footnote	—	0.024 *

All models were fitted to the same 1561 NRS assessments from 429 patients (Gaussian family with identity link, exchangeable working correlation, robust sandwich standard errors, patient as the clustering unit; estimated within-patient correlation 0.19). Mean difference refers to the NRS score associated with the exposure, in points. Model 1 is the primary specification and is the one reported in the Abstract; Model 2 is a secondary specification that additionally adjusts for age and treating institution as potential confounders. * Model 4 is a post hoc analysis reporting the Wald test for the interaction between discontinuation status and assessment time (χ^2^ = 9.46, df = 3); the corresponding time-specific mean differences associated with prior early discontinuation were +0.33 (95% CI −0.21 to +0.87) at 6 h, +0.53 (−0.06 to +1.12) at 12 h, +0.23 (−0.21 to +0.67) at 24 h, and −0.34 (−0.71 to +0.03) at 48 h, none of which was statistically significant. CI, confidence interval; CWI, continuous wound infiltration; IV-PCA, intravenous patient-controlled analgesia; NRS, numeric rating scale.

## Data Availability

The data presented in this study are available from the corresponding author upon reasonable request. The data are not publicly available due to privacy and ethical restrictions.

## Abbreviations

The following abbreviations are used in this manuscript:

CWI	continuous wound infiltration
IV-PCA	intravenous patient-controlled analgesia
LOS	length of stay
NRS	numeric rating scale
PONV	postoperative nausea and vomiting
SPA	single-port access

## Appendix A

**Table A1:** Sensitivity analysis of postoperative pain according to the timing of IV-PCA discontinuation.

Comparison (NRS Assessment)	Discontinued	Maintained	*p*-Value
6 h—discontinuers still on IV-PCA at 6 h (*n* = 38)	3.24 ± 1.13	3.04 ± 1.37	0.217
12 h—discontinued before 12 h (*n* = 26)	2.69 ± 1.57	2.18 ± 1.25	0.087
24 h—discontinued before 24 h (*n* = 46)	2.37 ± 1.48	2.13 ± 1.39	0.162
48 h—discontinued before 48 h (*n* = 48)	0.96 ± 1.17	1.22 ± 1.40	0.261
12 h—discontinued within 6 h (*n* = 17)	3.00 ± 1.41	2.18 ± 1.25	0.008 *
24 h—discontinued within 6 h (*n* = 20)	2.45 ± 1.76	2.13 ± 1.39	0.351

Values are mean ± SD; comparisons use the Mann–Whitney U test. Except for the first (pre-discontinuation reference) row, each comparison is restricted to NRS assessments obtained after IV-PCA discontinuation. The *n* for each row reflects two successive restrictions: patients meeting the timing condition for that row and, among them, those with an available NRS score at that time point. The corresponding counts (timing-eligible → missing NRS → analyzed) were: 6 h still receiving IV-PCA, 39 → 1 → 38; 12 h, 30 → 4 → 26; 24 h, 47 → 1 → 46; 48 h, 59 → 11 → 48; 12 h among those discontinuing within 6 h, 20 → 3 → 17; 24 h among the same subgroup, 20 → 0 → 20. One of the 60 discontinuers had an out-of-range discontinuation-timing record and was excluded from all timing-based rows (Figure 2); that patient also had no 48-h NRS assessment. The maintained comparison group comprised 371, 300, 348, and 329 patients with available assessments at 6, 12, 24, and 48 h, respectively. Timing categories use the convention ≤6 h, >6–24 h, >24–48 h. Subgroups are small, and this analysis is exploratory. * *p* < 0.05. IV-PCA, intravenous patient-controlled analgesia; NRS, numeric rating scale; SD, standard deviation.

**Table A2:** Relationship between the present analysis and previous publications from the same institutions.

	Kang et al. 2023 [12]	Kang et al. 2024 [13]	Present Analysis
Recruitment period	January 2020–August 2022	March 2020–September 2023	March 2020–September 2023
Patients screened	431 receiving IV-PCA or CWI	559 receiving IV-PCA or CWI	461 receiving IV-PCA or CWI
Received CWI alone (no IV-PCA)	0	109	30 (excluded; outside study population)
Started IV-PCA	431	450	431
Discontinued IV-PCA	60 (excluded from analysis)	89 (excluded from analysis)	60 (analyzed; primary outcome)
Maintained IV-PCA	371 (analyzed)	361 (analyzed)	371 (analyzed)
Focus of analysis	CWI + IV-PCA vs. IV-PCA alone, among patients maintaining IV-PCA	CWI alone vs. IV-PCA alone vs. combination; among IV-PCA recipients, only those maintaining IV-PCA were analyzed	Incidence, reasons, timing and correlates of IV-PCA discontinuation
Patient-level overlap with present analysis	Not verifiable (see footnote)	Not verifiable (see footnote)	—

Figures for the previous studies are those reported in the respective publications. Because each publication used a separate dataset extraction tied to its own inclusion criteria and analysis date, and no common patient identifier was retained across the extractions, the number of individual patients shared between studies could not be reconstructed and is reported as not verifiable. We note that the number of IV-PCA recipients (431) and of discontinuations (60) in the present analysis is identical to that reported in [12]; whether these represent the same individuals could not be confirmed at the patient level. The statement that the 60 discontinuers were not analyzed in either previous study is based on the published exclusion criteria of those studies (both excluded patients who did not maintain IV-PCA), not on a patient-by-patient comparison. IV-PCA, intravenous patient-controlled analgesia; CWI, continuous wound infiltration.

**Table A3:** Complete-case sensitivity analysis of factors associated with IV-PCA discontinuation.

Variable	Adjusted OR	95% CI	*p*-Value
Age (per year)	1.02	0.99–1.06	0.116
Body mass index (per kg/m^2^)	0.95	0.87–1.03	0.259
Previous abdominal surgery	1.13	0.60–2.09	0.707
Oophorectomy (vs. cystectomy)	0.99	0.42–2.25	0.977
Operative time (per min)	1.01	0.99–1.02	0.622
Concurrent CWI	1.22	0.67–2.21	0.519
Institution: Uijeongbu Eulji Medical Center	0.29	0.03–1.23	0.101

Firth-penalized logistic regression restricted to patients with complete covariate data (*n* = 419; 51 events); the corresponding primary analysis using multiple imputation is in Table 2. Reference categories are as in Table 2. Nine of the 10 discontinuation events at Uijeongbu Eulji Medical Center were lost due to missing body mass index data, which accounts for the instability of the institution estimate in this analysis. CI, confidence interval; CWI, continuous wound infiltration; IV-PCA, intravenous patient-controlled analgesia; OR, odds ratio.

**Table A4:** Postoperative NRS scores according to IV-PCA status, stratified by concurrent continuous wound infiltration.

Stratum	Time Point	Discontinued	Maintained	*p*-Value
No CWI	6 h	3.50 ± 1.14 (*n* = 30)	3.08 ± 1.34 (*n* = 212)	0.038 *
	12 h	2.63 ± 1.36 (*n* = 27)	2.14 ± 1.16 (*n* = 181)	0.059
	24 h	2.23 ± 1.33 (*n* = 30)	2.11 ± 1.44 (*n* = 196)	0.285
	48 h	0.71 ± 0.81 (*n* = 24)	1.34 ± 1.51 (*n* = 182)	0.056
Concurrent CWI	6 h	3.17 ± 1.10 (*n* = 29)	2.99 ± 1.40 (*n* = 159)	0.336
	12 h	2.50 ± 1.53 (*n* = 24)	2.24 ± 1.38 (*n* = 119)	0.364
	24 h	2.61 ± 1.47 (*n* = 28)	2.15 ± 1.32 (*n* = 152)	0.070
	48 h	1.21 ± 1.41 (*n* = 24)	1.08 ± 1.23 (*n* = 147)	0.721

Values are mean ± SD. Groups were compared within each stratum using the Mann–Whitney U test; these are unadjusted, exploratory post hoc comparisons in small subgroups and were not corrected for multiple testing. * Unadjusted *p* < 0.05. CWI, continuous wound infiltration; IV-PCA, intravenous patient-controlled analgesia; NRS, numeric rating scale; SD, standard deviation.
